# Testing times for dementia: a community survey identifying contemporary barriers to risk reduction and screening

**DOI:** 10.1186/s13195-023-01219-4

**Published:** 2023-04-10

**Authors:** Nikki-Anne Wilson, Ruth Peters, Nicola T. Lautenschlager, Kaarin J. Anstey

**Affiliations:** 1 Dementia Centre for Research Collaboration, Sydney, Australia; 2 Neuroscience Research Australia, Margarete Ainsworth Building, 139 Barker Street, Randwick, Sydney, NSW 2031 Australia; 3 School of Psychology, The University of New South Wales, Randwick, Sydney, Australia; 4 The George Institute for Global Health, Newtown, Sydney, Australia; 5 Academic Unit for Psychiatry of Old Age, Department of Psychiatry, The University of Melbourne, Parkville, Melbourne, Australia; 6 North Western Mental Health, Royal Melbourne Hospital, Parkville, Melbourne, Australia

**Keywords:** Risk reduction, Dementia, Dementia prevention, Dementia testing, Population health, Health Screening, Barriers and enablers

## Abstract

**Background:**

Advances in pharmacological and non-pharmacological dementia interventions may mean future dementia prevention incorporates a combination of targeted screening and lifestyle modifications. Elucidating potential barriers which may prevent community engagement with dementia prevention initiatives is important to maximise the accessibility and feasibility of these initiatives across the lifespan.

**Methods:**

Six hundred seven adults aged over 18 years completed a 54-item, multiple-choice survey exploring contemporary attitudes towards, and barriers to, dementia risk reduction and screening relative to other common health conditions. Participants were sourced from Australia’s largest, paid, data analytics service (ORIMA).

**Results:**

Finances (*p* = .009), poor motivation (*p* = .043), and time (*p* ≤ .0001) emerged as significant perceived barriers to dementia risk reduction behaviours. Lack of time was more likely to be reported by younger, relative to older, participants (*p* ≤ .0001), while females were more likely than males to report financial (*p* = .019) and motivational (*p* = .043) factors. Binary logistic regression revealed willingness to undertake dementia testing modalities was significantly influenced by gender (genetic testing, *p* = .012; saliva, *p* = .038, modifiable risk factors *p* = .003), age (cognitive testing, *p* ≤ .0001; blood, *p* = .010), and socio-economic group (retinal imaging, *p* = .042; modifiable risk-factor screening, *p* = .019). Over 65% of respondents felt adequately informed about risk reduction for at least one non-dementia health condition, compared to 30.5% for dementia.

**Conclusions:**

This study found perceived barriers to dementia risk reduction behaviours, and the willingness to engage in various dementia testing modalities, was significantly associated with socio-demographic factors across the lifespan. These findings provide valuable insight regarding the accessibility and feasibility of potential methods for identifying those most at risk of developing dementia, as well as the need to better promote and support wide-scale engagement in dementia risk reduction behaviours across the lifespan.

**Supplementary Information:**

The online version contains supplementary material available at 10.1186/s13195-023-01219-4.

## Introduction

Equitable and effective dementia risk reduction necessitates both an individual and population focused approach [1]. The global cost of dementia is currently estimated as US$818 billion and dementia is a World Health Organisation (WHO) public health priority [2]. Dementia prevention research aims to reduce this burden, and associated personal costs, by identifying risk factors and implementing effective risk reduction initiatives which both support population health and target those most in need [3]. Advances in biomarker research provide a growing potential for the implementation of population based dementia screening initiatives as part of this risk reduction strategy. Despite screening programs historically being deemed ineffective and/or unfeasible [4], the future of such initiatives show promise for identifying and supporting those most at risk to adopt behaviour changes earlier in the life course [1]. One’s capability, motivation, and opportunity to enact positive behaviour change [5], however, are dependent on a range of socio-demographic factors. For the purposes of this study, we focus on three key social determinants of health as identified by the World Health Organization [6] representing individual characteristics (gender, age) and socio-economic context. Here, we use a twofold approach to reveal how these key socio-demographic factors may impact dementia risk reduction. First, we elucidate community knowledge and understanding of dementia risk reduction, and second, we identify perceived barriers experienced across socio-demographic groups which may limit engagement in risk reduction behaviours, including willingness to undertake dementia screening.

### The dementia risk reduction landscape

Recent decades have seen the identification of a plethora of factors occurring across the life-course which likely increase dementia risk [7]. These risk factors exist, to varying degrees, across socio-economic and cultural groups [8], highlighting the need for an inclusive approach to dementia prevention. The dementia prevention landscape, however, is complex with many of the factors identified requiring both an individual and/or population-level approach to best support effective positive behavioural change. Recent WHO evidence-based recommendations for reducing dementia risk across the life-span include the following: increasing physical activity; healthy diet, particularly a Mediterranean-like diet; avoidance of mid-life obesity; prevention and management of hypertension, dyslipidaemia and diabetes; smoking cessation; reducing hazardous alcohol consumption; and engaging in cognitive training/stimulation [2]. Collectively, these findings speak to the need to identify broad barriers to community engagement in dementia preventative behaviours, now and in the future.

### Risk reduction barriers—lack of awareness and knowledge

Currently, the paucity of disease modifying pharmaco-therapeutic approaches in dementia exists within the context of an evolving, whole-of-life and lifestyle approach to dementia prevention [7]. Identifying ways to effectively reach those most in need of up-to-date dementia risk reduction information across the lifespan is imperative to avoid community confusion and disengagement [9, 10]. Globally, knowledge of dementia and its risk factors remains poor [11, 12]. Respondents failed to identify six out of ten empirically supported modifiable risk factors in a recent study [13], and 20% of young adults (aged 18–44 years) failed to identify any modifiable risk factors for dementia at all [14]. Considering some dementia risk factors are more likely to be incurred in young adulthood, e.g. (mild) traumatic brain injury [15] and hearing damage [16], there is an increasing need to have dementia risk reduction awareness breakthrough across generations. Knowledge of ways to support cognitive health is also reported to be significantly influenced by socio-economic factors [17] and social determinants of health are becoming of progressively greater importance in dementia risk reduction [18]. The social, economic, and environmental context in which people make lifestyle choices is imperative to achieve successful long-term behaviour change [10]. Individual characteristics (e.g. age, gender) and attitude also play a role in risk reduction behaviour [5, 19], and willingness to engage in dementia preventative behaviours is significantly influenced by, among other factors, one’s perceived susceptibility [20].

### Risk reduction barriers—willingness to undertake dementia testing

The UK failed to recommend a national dementia screening program in 2019 based on ambiguous extant evidence for cognitive assessment tools, unclear progression from detection of mild cognitive impairment to dementia, and a lack of reliable biomarkers [4]. Considerable advancements continue to be made, however, and the rapidity of development of biological measures for determining dementia risk, for example, genetic testing [21], blood, and cerebrospinal fluid (CSF) [22] requires an equally progressive understanding of how these developments may best be utilised. The seeming importance of identifying those at greatest risk for dementia, particularly those likely to progress from a diagnosis of mild cognitive impairment [23], is also advancing at a considerable pace and will likely play an important part in the future of dementia risk reduction, at least at an individual level. In conjunction with broader prevention strategies, dementia screening has the potential to increase the ability to effectively target and support at-risk individuals earlier in the life-course [24]. Despite previous reports identifying considerable hesitancy and broad concerns [25] regarding these tests within the community, and among physicians [25] and dementia caregivers [26], it is important that research in this area continues to keep pace with overall scientific advancements and community perceptions to maximise potential future benefits. Ascertaining perceived barriers and enablers regarding biomarker screening is imperative if we are to ensure a viable and ethical approach to the collection of these measures. Responsible assessment of an individual’s dementia susceptibility will continue to require an integrative approach for the foreseeable future, incorporating both the broader clinical picture and potential biomarkers. In conjunction with appropriate support [27], however, early reports in limited samples offer promising evidence that there may be a place for dementia screening within the context of family decision-making and clinical trials [28].

### Study aims

This study aimed to examine the contribution from three key socio-demographic factors—age, gender, and socio-economic group—which may increase the likelihood of experiencing barriers to dementia risk reduction, including one’s willingness to engage in various dementia testing modalities. First, we examined how knowledge of dementia risk reduction compares to other common health conditions. Second, we examined socio-demographic barriers to engaging in dementia risk reduction, including dementia testing. Based on the previously recognised impact from these factors on behaviour change [19], we hypothesised age, gender, and socio-economic group would significantly influence the perceived feasibility of engaging in dementia risk reduction behaviours, including willingness to engage in dementia testing.

## Methods and materials

### Participants

All participants were aged over 18 years, and ability to take part in the online survey was taken as evidence of technological and English proficiency. A total of 607 respondents completed the nation-wide survey, delivered by Australia’s largest data analytics service (ORIMA) from a pool of 452,000 registered users. For a full overview of the demographic profile of ORIMA users in relation to the Australian population, please see Supplementary Material A (Table S1). In brief, the ORIMA pool of registered users’ age, gender, and location (state/territory) are comparable to the Australian population. The ORIMA registry does not collect educational attainment; however, data from the current sample can be seen in Table 1. Of the 672 ORIMA users who clicked the survey link, 90% completed the survey, and seven participants failed the screener due to being under 18. No identifying data were collected and participation was voluntary. A small financial incentive (maximum AU$2.00) based on estimated survey completion time was provided to registered participants. The study was approved by the University of New South Wales Human Research Ethics Advisory Panel (HC 3508).

**Table 1:** Demographic characteristics of the survey sample

	18–39 *n*	40–59 *n*	60 + *n*	Total *n*	Total %
Gender					
Male	131	82	86	299	49.4
Female	99	109	96	304	50.3
Other	1	-	1	2	0.3
Cultural background					
Australian born	188	149	135	472	77.8
Aboriginal and/or Torres Strait Islander	10	2	1	13	2.1
Other ethnicity	32	33	24	89	14.7
Speaks English at home	188	169	174	531	87.5
Location					
New South Wales	84	51	61	196	32.3
Victoria	57	60	40	157	25.9
Queensland	38	42	41	121	19.9
South Australia	16	15	10	41	6.8
Western Australia	26	16	19	61	10.0
Australian Capital Territory	4	3	6	13	2.1
Northern Territory	4	-	2	6	1.0
Tasmania	3	5	4	12	2.0
Regional	38	51	78	167	27.5
Metropolitan	194	141	105	440	72.5
Education					
High-school or below	39	65	70	174	28.7
Technical/trade certificate	22	35	44	101	16.6
Bachelor degree	125	53	28	206	33.9
Masters and/or doctorate	26	18	7	51	8.4
Employment and living					
Currently employed	183	121	25	329	54.2
Seeking work	15	21	4	40	14.4
Live alone	46	46	65	157	26.2
Low SES	33	46	41	120	19.8
Average SES	89	55	75	219	36.2
High SES	110	91	65	266	44.0
Dementia connection					
Person with MCI	6	3	2	11	1.8
Person with dementia	3	2	-	5	0.8
I am a carer for a PWD	14	6	4	24	4.0
Relative/friend of a PWD	47	46	48	141	23.2
Health professional	20	4	2	26	4.3
No direct connection	141	131	127	399	65.7

*SES* socio-economic status, *MCI* mild cognitive impairment, *PWD* person with dementia

### Measures

All data was collected via an online survey curated by the research team and based on a broad representation of factors relating to the behaviour change model of health interventions [5], including, motivation, opportunity, and capability. Incorporating questions representing each of these facets of health behaviour, the survey comprised 54 multiple-choice questions broken into three main sections (plus demographics) for ease of completion. Questions pertained to dementia and other common health conditions (heart disease, stroke, cancer, and mental illness). Survey sections and example questions are described below.

#### Demographics

Age range, gender, years of education, employment, and information pertaining to language and cultural background were collected to determine the representativeness of the sample.

#### Lifestyle habits and disease prevention

This section included questions pertaining to the level of engagement in, and knowledge of, dementia risk reduction relative to other health conditions (heart disease, stroke, cancer, mental illness). Example questions included, “Do you feel adequately informed about the ways in which you can reduce your risk of developing health problems?”, “Do you feel that information about reducing your risk of developing health problems is easily available?”, “From which sources do you/would you seek information about reducing your risk of developing health problems?”, “Do you feel confident in your ability to apply healthy lifestyle strategies to your overall health management to help prevent serious disease?”.

#### Health testing

As an extension of understanding respondents’ motivation to engage in dementia risk reduction, this section pertained to willingness to undertake health screening. Example questions include, “If you had the option, would you like to know your likelihood of developing dementia?”, “Do you feel that knowing your likelihood of developing dementia may influence your plans for the future?”, “If it was affordable, would you be willing to pay for testing in order to determine your risk of developing dementia?”, “Which of the following assessments would you be willing to undertake in order to determine your likelihood of developing dementia?”.

#### Dementia knowledge

In order to determine the level of understanding of dementia, as well as ascertain respondents’ overall lived experience of dementia, this section included general questions regarding the various facets of dementia and any connection to those living with the disease. Example questions include: “Which of the following symptoms do you associate with a dementia diagnosis?”, “Of which of the following forms of dementia are you aware?”, “Which of the following best describes your connection to dementia?”.

#### Socio-economic status

Socio-economic status was measured using the Socio-Economic Indexes for Areas (SEIFA) according to the Australian Bureau of Statistics (ABS) decile ranking of postal areas (POAs) [29]. Based on the Australian Statistical Geography Standard (ASGS), the SEIFA ranks areas in Australia according to relative socio-economic advantage and disadvantage. Using decile rankings, participants were divided into low (≤ 3), average (4–7), and high (≥ 8) socio-economic groups.

### Procedure

The survey was distributed via Orima. Participants were sent an email and invited to click the link to complete the survey which was conducted fully online. A brief screener was completed prior to the survey to determine eligibility based on age and country of residence. Questions were then displayed one at a time with progression dependent on answering the previous question. A “don’t know” response was included where appropriate to allow participants to bypass a particular question while maximising survey completion.

### Statistical analyses

Statistical analyses were conducted in IBM SPSS version 26. Descriptive statistics are displayed as number of participants endorsing the response and/or percentages. Due to limited sample size, it was not possible to include all demographic factors in the analyses, rather, all models were informed by the study aims. Binary logistic regression models and odds ratios were used to examine these demographic factors (age, gender, socio-economic status) and whether they increase the likelihood of reporting perceived barriers to dementia risk reduction or willingness to undertake screening. Chi-square goodness of fit and percentage accuracy of classification (PAC) scores were used to assess model-fit. Due to the relatively limited sample size, Bonferroni correction for multiple comparisons was deemed overly conservative; therefore, confidence intervals and standardised beta coefficients are included to aid the reader’s interpretation of model fit. All uncorrected results are reported using a significance threshold of *p* < 0.05.

## Results

### Sample characteristics

Distribution of age (18–39, 38.2%; 40–59, 31.6%; 60 + , 30.1%) and gender (49.4% male) were largely consistent with the broader Australian population [30] (Table 1 and Supplementary Material A, Table S1). Only two non-binary people participated and due to amalgamation of this group within the other two gender categories being inappropriate, only participants identifying as male or female were included in gender analyses. Participants reported a substantially higher level of educational attainment (33.9% bachelors) relative to the Australian population (24% bachelors [29]). The majority (65.7%) reported no direct connection to dementia (e.g. family member; health professional).

### Dementia knowledge

Dementia was reported as a natural part of ageing by 20.1% of respondents, although this decreased with age (18–39, 27.2%; 40–59, 19.3%; 60 + , 12.0%). Alzheimer’s was the most identified form of dementia (70.5%), followed by vascular (15.5%), dementia with Lewy bodies (14.3%), and frontotemporal (11.5%). Over a quarter (25.9%) reported being unaware of any of the dementias listed; however, this decreased with age (18–39, 31.9%; 40–59, 29.2%; 60 + , 14.8%). Memory issues were identified by 85.0% as a sign of dementia, followed by getting lost (75.5%), difficulty planning/organising (62.8%), and loss of social skills (58.2%). Losing interest in things once enjoyed and showing less emotion were each identified by 51.4%, while only 31.1% recognised perceptual difficulties as indicative of dementia.

### Dementia risk reduction—barriers and enablers

**Fig. 1:**
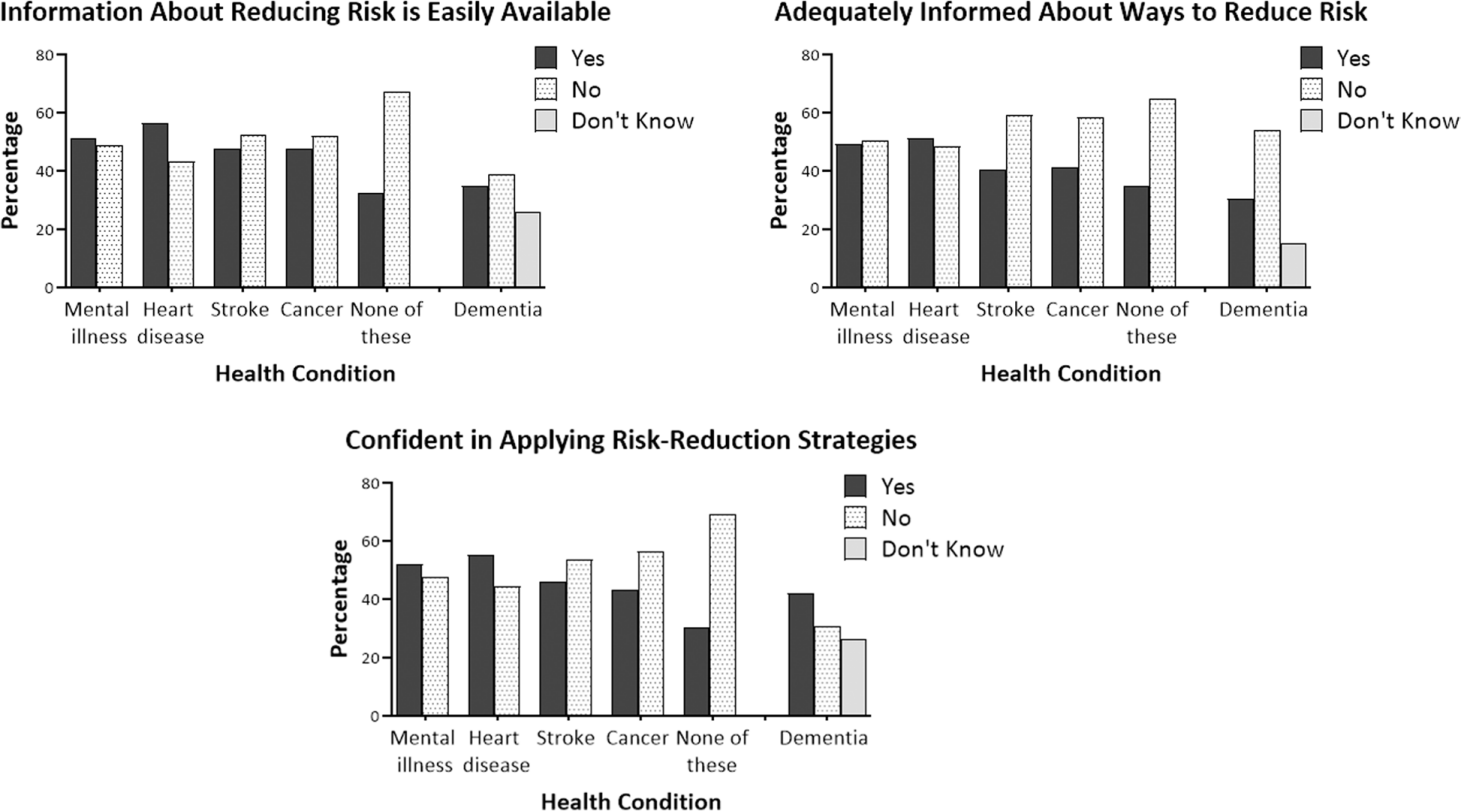
Percentage of respondents endorsing items for non-dementia health conditions and dementia. ‘Don’t know’ response included in dementia item only. See Supplementary Material B for further information pertaining to individual survey items

Participants were generally less informed about reducing dementia risk compared to risk reduction for other conditions (Fig. 1). Over 65% felt adequately informed about risk reduction for at least one non-dementia health condition compared to 30.5% for dementia. Information about dementia risk reduction was also perceived as less available compared to other health conditions and this was echoed by a reduced level of confidence in applying dementia risk reduction (Fig. 1). Perceived self-efficacy (i.e. level of control) in reducing risk was also lower for dementia (34.2%) compared to general health (54.9%). Only 10.0% identified early-life factors as contributing to dementia risk, with all age-groups reporting middle-age as the most important life-stage for dementia risk reduction (18–39, 35.3%; 40–59, 41.1%; 60 + , 50.3%). The priority of dementia risk reduction increased with age (18–39, 25.4%; 40–59, 30.2%; 60 + , 38.8%).

Binary logistic regression revealed, with other variables held constant, older participants (60 +) were 2.4 times more likely to report being adequately informed about dementia risk reduction relative to younger participants (18–39), nearly 2.3 times more likely to report this information as being easily available, and approximately 3.6 times more likely to feel confident in applying dementia risk reduction. Females were less likely than males to consider dementia risk as under their control, while older participants (60 +) reported feeling more in control of their risk of developing dementia than younger participants (Table 2).

**Table 2:** Knowledge and awareness of dementia risk reduction and demographic variables

			*B*	SE	*p*	OR	CI (95%)		*χ*^2^	*p*	PAC		
							Lower	Upper			Constant		Model
Adequately informed about dementia risk reduction									22.87	.**000**	64.1		65.7
% Yes 30.5	Gender (male)		− 0.03	0.19	.896	0.98	0.67	1.42					
	Age group (18–39)				.**000**								
		40–59	− 0.05	0.24	.824	0.95	0.60	1.51					
		60 +	0.88	0.23	.**000**	2.40	1.54	3.74					
	Socio-economic status (low)				.538								
		Average	− 0.04	0.27	.887	0.96	0.57	1.62					
		High	− 0.24	0.26	.361	0.79	0.48	1.31					
Information about dementia risk reduction is easily available									14.39	.**013**	52.9		58.6
% Yes 34.8	Gender (male)		− 0.01	0.20	.962	0.99	0.68	1.45					
	Age group (18–39)				.**002**								
		40–59	0.26	0.23	.260	1.30	0.82	2.05					
		60 +	0.82	0.24	.**001**	2.28	1.43	3.64					
	Socio-economic status (low)				.506								
		Average	− 0.04	0.28	.878	0.96	0.56	1.65					
		High	− 0.25	0.27	.338	0.78	0.46	1.30					
Confident in ability to apply dementia risk reduction									31.28	.**000**		57.5	61.3
% Yes 42.3	Gender (male)		− 0.08	0.20	.681	0.92	0.62	1.37					
	Age group (18–39)				.**000**								
		40–60	0.40	0.24	.094	1.49	0.94	2.38					
		60 +	1.27	0.25	.**000**	3.55	2.18	5.80					
	Socio-economic status (low)				.479								
		Average	− 0.04	0.29	.889	0.96	0.55	1.69					
		High	− 0.27	0.28	.333	0.76	0.44	1.32					
Likelihood of developing dementia somewhat under your control?									20.71	.**001**		51.2	57.7
% Yes 34.2	Gender (male)		− 0.60	0.20	.**003**	0.55	0.37	0.82					
	Age group (18–39)				.**003**								
		40–60	0.35	0.25	.158	1.42	0.87	2.31					
		60 +	0.83	0.25	.**001**	2.29	1.42	3.71					
	Socio-economic status (low)				.611								
		Average	0.04	0.29	.899	1.04	0.59	1.82					
		High	− 0.18	0.27	.517	0.84	0.49	1.43					

Reference category shown in parentheses. *B*, unstandardized beta values. *SE* standard error, *OR* odds ratio, *CI* confidence interval, *PAC* percentage accuracy of classification. Gender representative of male and female only due to limited non-binary sample

More than half of respondents (61.8%) identified at least one barrier to dementia risk reduction. Lack of time (15.2%), motivation (29.5%), and affordability (21.3%, e.g. cost of gym membership, healthy food, or psychological treatment) were the most frequently reported barriers. With other variables held constant, those from a lower socio-economic background and females were more likely to report financial barriers to dementia risk reduction. Younger participants were more likely to report lack of time, and females more likely to cite poor motivation, as barriers to dementia risk reduction (Table 3).

**Table 3:** Barriers to dementia risk reduction and demographic variables

			*B*	SE	*p*	OR	CI (95%)		*χ*^2^	*p*
							Lower	Upper		
Financial									15.47	.**009**
% Yes 21.3	Gender (male)		0.49	0.21	.**019**	1.63	1.08	2.44		
	Age group (18–39)				.840					
		40–59	0.03	0.25	.895	1.03	0.64	1.67		
		60 +	− 0.11	0.25	.652	0.89	0.55	1.46		
	Socio-economic status (low)				.**014**					
		Average	0.30	0.27	.264	1.36	0.80	2.31		
		High	− 0.37	0.28	.194	0.69	0.40	1.20		
Lack of time									66.13	.**000**
% Yes 15.2	Gender (male)		0.07	0.24	.772	1.07	0.67	1.72		
	Age group (18–39)				.**000**					
		40–59	− 0.75	0.26	**.004**	0.47	0.28	0.79		
		60 +	− 3.06	0.60	**.000**	0.05	0.01	0.15		
	Socio-economic status (low)				.079					
		Average	0.91	0.40	.025	2.48	1.12	5.46		
		High	0.75	0.40	.058	2.11	0.97	4.57		
Lack of motivation									11.44	.**043**
% Yes 29.5	Gender (male)		0.37	0.18	**.043**	1.45	1.01	2.08		
	Age group (18–39)				.053					
		40–59	− 0.30	0.22	.160	0.74	0.48	1.13		
		60 +	− 0.54	0.23	**.017**	0.58	0.38	0.91		
	Socio-economic status (low)				.302					
		Average	− 0.10	0.25	.698	0.91	0.56	1.48		
		High	− 0.34	0.24	.164	0.71	0.44	1.15		

Percentage of total respondent endorsement shown in italics. Reference category in parentheses. *B*, unstandardized beta values. *SE*, standard error. *OR*, odds ratio. *CI*, confidence interval. Gender represents male and female only due to limited non-binary sample

Potential enablers for increasing dementia risk reduction include lowering the cost of healthy food (45.1%) and advice on healthy eating (30.8%). Ease of access to information (32.5%); community classes (25.9%, e.g. dance, fitness, drama); increased access to allied health (21.9%); more green space (20.6%); and greater incentives (24.5%) were also endorsed as likely increasing dementia risk reduction.

### Dementia screening—attitudes and concerns

Over half of respondents (62.6%) would like to know their dementia risk, 89.5% of whom would still want to know despite limited treatment options. Nearly half (48.4%) would consider seeing a professional to help decide whether dementia testing was appropriate, while 41.7% reported being aware that dementia testing may be inconclusive. Over half (51.1%) would be willing to pay for dementia testing if it were affordable. The majority (65.7%) reported knowing their dementia susceptibility would influence their lifestyle choices, of whom 93.7% reported this would make them more likely to make positive changes (Supplementary Material A, Table S2).

The most common reason cited for not wishing to know one’s dementia risk was “just rather not know” (42.7%), followed by anxiety (35.9%), and lack of ability to do anything about it (30.8%). Of note, 6.8% reported dementia testing as not in line with their cultural or religious values, while 13.7% reported not trusting the results. Repercussions for employment (20.3%), and relationships (23.2%) were the most common concerns regarding the implications of dementia testing, followed by obtaining insurance (18.6%), privacy (15.3%), and family planning (14.5%).

### Dementia screening—testing modalities

Considerable variability emerged in willingness to undertake dementia testing across modalities, with age, socio-economic group, and gender significantly influencing willingness to engage in particular forms of testing. Saliva testing was the most acceptable method of dementia testing endorsed by 59.9% of respondents, closely followed by blood testing (59.2%), genetic testing (57.5%), and cognitive testing (57.5%). For a full breakdown of willingness to undertake dementia testing by age and gender, see Supplementary Material A, Table S3. The relationship between willingness to undertake dementia testing modalities and demographic variables is examined in Table 4.

**Table 4:** Willingness to undertake dementia testing procedures and demographic variables

			*B*	SE	*p*	OR	CI (95%)		*χ*^2^	*p*	PAC	
							Lower	Upper			Constant	Model
Blood test									11.65	**.040**	59.2	59.7
(%Yes 59.2)	Gender (male)		0.20	0.17	.245	1.22	0.87	1.70				
	Age group (18–39)				**.025**							
		40–59	0.39	0.20	.056	1.47	0.99	2.19				
		60 +	0.53	0.21	**.010**	1.70	1.13	2.56				
	SES (low)				.204							
		Average	0.37	0.23	.114	1.45	0.92	2.29				
		High	0.37	0.23	.097	1.45	0.93	2.26				
Saliva test									20.80	**.001**	59.9	62.6
(%Yes 59.9)	Gender (male)		0.35	0.17	**.038**	1.43	1.02	1.99				
	Age group (18–39)				**.023**							
		40–60	0.50	0.21	**.015**	1.65	1.10	2.47				
		60 +	0.46	0.21	**.026**	1.59	1.06	2.39				
	SES (low)				**.011**							
		Average	0.70	0.24	**.003**	2.02	1.27	3.21				
		High	0.51	0.23	**.025**	1.66	1.06	2.58				
Genetic test									14.22	**.014**	57.6	59.6
(%Yes 57.5)	Gender (male)		0.43	0.17	**.012**	1.53	1.10	2.13				
	Age group (18–39)				.079							
		40–60	0.22	0.20	.286	1.24	0.84	1.84				
		60 +	0.47	0.21	**.024**	1.59	1.06	2.39				
	SES (low)				.378							
		Average	0.32	0.23	.166	1.38	0.87	2.19				
		High	0.23	0.23	.300	1.26	0.81	1.96				
Lumbar puncture									5.13	.400	88.0	88.0
(%Yes 11.9)	Gender (male)		− 0.35	0.26	.168	0.70	0.43	1.16				
	Age group (18–39)				.342							
		40–61	0.19	0.32	.547	1.21	0.65	2.26				
		60 +	0.45	0.31	.145	1.56	0.86	2.85				
	SES (low)				.483							
		Average	0.34	0.38	.379	1.40	0.66	2.96				
		High	0.44	0.37	.228	1.56	0.76	3.21				
Aroma test									6.95	.224	56.4	58.1
(%Yes 43.6)	Gender (male)		0.28	0.17	.093	1.32	0.95	1.84				
	Age group (18–39)				.273							
		40–62	0.27	0.20	.174	1.32	0.89	1.95				
		60 +	0.29	0.20	.163	1.33	0.89	1.98				
	SES (low)				.515							
		Average	0.05	0.23	.837	1.05	0.66	1.66				
		High	0.22	0.23	.326	1.25	0.80	1.94				
Retinal imaging									14.91	**.011**	50.1	56.4
(%Yes 49.9)	Gender (male)		0.28	0.17	.091	1.33	0.96	1.84				
	Age group (18–39)				.052							
		40–63	0.36	0.20	.075	1.43	0.96	2.12				
		60 +	0.47	0.20	.022	1.59	1.07	2.38				
	SES (low)				**.042**							
		Average	0.55	0.23	**.019**	1.73	1.09	2.74				
		High	0.51	0.23	**.025**	1.66	1.07	2.59				
Cognitive testing									20.18	**.001**	57.6	60.4
(%Yes 57.5)	Gender (male)		0.31	0.17	.067	1.36	0.98	1.90				
	Age group (18–39)				**.001**							
		40–64	0.23	0.20	.257	1.26	0.85	1.86				
		60 +	0.79	0.21	**.000**	2.20	1.46	3.33				
	SES (low)				.479							
		Average	0.28	0.24	.238	1.32	0.83	2.09				
		High	0.23	0.23	.321	1.25	0.80	1.95				
Physiological testing									2.70	.746	60.4	60.4
(%Yes 39.6)	Gender (male)		0.07	0.17	.693	1.07	0.77	1.49				
	Age group (18–39)				.384							
		40–65	− 0.07	0.20	.732	0.93	0.63	1.39				
		60 +	− 0.28	0.21	.176	0.76	0.50	1.13				
	SES (low)				.741							
		Average	0.18	0.24	.443	1.20	0.75	1.91				
		High	0.10	0.23	.662	1.11	0.71	1.73				
Modifiable risk factors									17.67	**.003**	55.7	57.6
(%Yes 44.3)	Gender (male)		0.51	0.17	**.003**	1.66	1.19	2.31				
	Age group (18–39)				.143							
		40–66	− 0.07	0.20	.735	0.93	0.63	1.39				
		60 +	0.32	0.20	.114	1.38	0.93	2.06				
	SES (low)				.064							
		Average	0.39	0.24	.106	1.47	0.92	2.35				
		High	0.54	0.23	**.019**	1.72	1.09	2.70				

*SES* socio-economic status, *B* beta value, *SE* standard error, *OR* odds ratio, *CI* 95% confidence interval, *PAC* percentage accuracy in classification. Reference category shown in parentheses. Gender represents male and female only due to limited non-binary sample

## Discussion

This study highlights perceived barriers to engaging in dementia risk reduction and testing which significantly vary across age, gender, and socio-economic groups. Knowledge of dementia risk factors, the ease with which this information is perceived to be available, and the level of confidence in applying risk reduction strategies were all poorer relative to other common health issues. Over half of respondents would like to know their risk of developing dementia, indicating increasing community acceptance relative to previous studies [25, 26]. Within the context of advancing understanding of the biological and lifestyle risk factors for dementia, here, we discuss the accessibility and feasibility of future wide-scale dementia risk reduction initiatives across key socio-demographic groups.

### Dementia risk reduction—barriers and enablers

Significant barriers to engaging in dementia risk reduction were found, particularly lack of motivation, and financial limitations. Females were more likely to report poor motivation; however, previous findings indicate individual differences within genders are likely more diverse than differences between genders, at least in older adults [31]. Considering women continue to disproportionately shoulder the burden of unpaid domestic work, this “mental load” has been suggested to negatively affect women’s overall health and motivation [32]. Motivation to engage in positive health behaviours may also be impacted by one’s level of perceived control regarding the effectiveness of one’s own actions [33], with males more likely than females to report the probability of developing dementia as somewhat under their control. The priority of dementia risk reduction also understandably increased with age, speaking to likely competing demands for time and resources experienced by younger adults and the closer proximity for older adults to the age at which the disease is likely to develop.

Socio-economic status significantly influenced the likelihood of reporting financial barriers to dementia risk reduction, with females and those from lower socio-economic areas experiencing greater financial hindrance. Nevertheless, fiscal themes emerged across study groups with nearly half of respondents (45.1%) reporting lowering the cost of healthy food as increasing their likelihood to engage in dementia risk reduction. Lower socio-economic groups already bear the brunt of higher living costs with affordable, healthful food an important issue for low-income households around the world [34, 35]. It must be acknowledged that equitable solutions to these perceived barriers will require systemic change at the population level and necessitate significant government expenditure. Increased accessibility to allied health services, community dance or fitness classes, and greater incentives were all endorsed as likely increasing engagement in dementia risk reduction, indicating an existing demand for a systemic approach. Further, the economic burden from dementia itself will likely continue to escalate [2]; therefore, prioritising population-level initiatives which support positive behavioural change across the lifespan will be needed to curb this emerging health and economic crisis.

### Dementia risk reduction relative to other health conditions

Community knowledge of dementia risk reduction and the confidence to prevent the development of dementia were lacking in comparison to cancer, cardiovascular disease, and mental health, particularly in younger people. This is important considering dementia outranks the majority of other health issues (second only to ischemic heart disease) as leading cause of death [30]. While many of the known lifestyle modifications associated with reducing dementia risk overlap with cardiovascular disease or cancer, for example, high blood pressure [36], some do not, for instance hearing loss and early life education [37]. Due to the complexity of dementia risk reduction relative to other health conditions, and the need to address various risk factors across the lifespan, a more targeted approach will become progressively more important as we move to reduce dementia risk at both the individual and population level.

### Risk reduction—acceptance of dementia screening

The majority of respondents reported that they would want to know their dementia risk, nearly 90% of whom would still want to know even in light of limited treatment options, indicating attitudes towards dementia screening may be evolving from previous lackluster support [25, 38]. Respondents were largely willing to pay for dementia testing, potentially important for future economic modelling regarding the feasibility of dementia screening but also highlighting the potential for such measures to increase existing inequities [39]. Considerable variability, however, was noted in acceptable screening modalities with community support to undertake more invasive testing (e.g. lumbar puncture), not unexpectedly, particularly lacking. Given cerebrospinal fluid has shown promise for current dementia diagnostic accuracy and screening [22, 40], moving away from invasive measures, or providing more education and support surrounding the procedure, will likely be necessary for wide-spread community uptake of any future screening initiatives [41]. Blood and saliva were more acceptable measures of dementia risk and there are encouraging advances being made in the utility of these less invasive methods [42].

Demographic factors significantly influenced willingness to engage in dementia testing, with higher socio-economic groups more likely to undertake retinal imaging and assessment of modifiable risk factors than lower socio-economic groups, possibly speaking to the perceived accessibility of these methods in the face of financial hardship. More familiar measures, such as blood tests, were more acceptable across socio-economic groups. Gender also influenced the palatability of assessment, with females more willing to undertake saliva, genetic, and modifiable risk-factor screening compared to males, with genetic screening potentially more salient for females due to concerns regarding family planning. Finally, older-age significantly increased willingness to undergo cognitive testing, with increased anxiety concerning declining memory [43] and the proximity to the life-stage at which dementia is likely to develop possibly contributing to this result.

### Risk reduction—concerns regarding dementia screening

Repercussions for employment and relationships particularly emerged as potential concerns regarding dementia screening, as well as anxiety and lack of trust regarding the results, supporting previously identified trepidations [25]. Importantly, our finding that nearly half of respondents would consider seeking professional support to make the decision whether or not to screen for dementia supports the need for more clinical training in this area and a multi-disciplinary approach. Encouragingly, the majority of participants reported that the ability to determine one’s dementia risk would likely result in positive lifestyle changes. Further investigation of the utility of identifying individuals at risk for dementia in order to support positive behavioural change will be needed, however, as knowledge of health risk alone may not always translate to positive health outcomes, e.g. lung cancer and smoking cessation [44]. Responsibly informing individuals of their dementia risk and providing appropriate individual and systemic support to enact positive behavioural change will likely play an increasing role in addressing global dementia risk reduction in coming decades.

### Study limitations

As with all survey data, some caution is warranted based on the representativeness of the sample, with survey participation more likely when the respondent is interested in the topic [45]. Of note, the online delivery of the study precludes many marginalised people in the population without internet access from participating and our sample was limited in culturally and linguistically diverse and Aboriginal and Torres Strait Islander people, likely underrepresenting diverse cultural perspectives. Due to limitations in time and study funding, it was not possible to conduct in-person/postal assessment; however, this is an important consideration regarding how our results may inform population level dementia prevention initiatives. Considering those with internet access reported difficulty finding information about dementia risk reduction, it is likely limited internet access would merely exacerbate this result. Broader representation of the overall experiences of those from minority groups is needed, however, to enhance discussions regarding how to equitably address perceived barriers to dementia risk reduction. As an averaged index of socio-economic advantage and disadvantage, the representativeness of the SEIFA may also be limited. Finally, we note our sample was highly educated, likely due to the online nature of the study, and this bias limited the ability to examine the role of this variable.

### Summary and conclusions

This study highlights the ongoing need to keep pace with contemporary attitudes and concerns pertaining to dementia risk reduction by identifying key barriers to the accessibility and feasibility of dementia prevention initiatives. We expand previous findings by highlighting acceptance of various dementia testing modalities and perceived barriers significantly vary across key socio-demographic factors, namely age, gender, and socio-economic group. Knowledge of dementia risk factors, and the level of confidence in applying risk reduction strategies, continues to lag behind other common health issues. This is important as prevention strategies for other health conditions may not fully capture the broad spectrum of known dementia risk factors across the lifespan [37]. The individual, community, and economic burden from dementia is predicted to exponentially increase over coming decades [3]. This study provides valuable insight regarding the need to consider individual and systemic barriers to engaging in dementia risk reduction behaviours in order to best support those most at risk.

## Supplementary Information

**Additional file 1:** **Supplementary Material A.** **Table S1.** Overview of source population (ORIMA) and comparable census demographics. **Table S2.** Percentage of respondents willing to undertake each dementia testing modality by age group and gender. **Table S3.** Percentage of respondents endorsing items pertaining to dementia testing by age group and gender. **Supplementary Material B.** Information pertaining to individual survey items.

## Data Availability

The datasets that support the findings of this study are available on request from the corresponding author. The data are not publicly available due to ethical restrictions.
