# Supplementary material for: Testing times for dementia: a community survey identifying contemporary barriers to risk reduction and screening
Source: Alzheimers Res Ther. 2023 Apr 10;15:76. doi: 10.1186/s13195-023-01219-4 (PMC10088195; doi:10.1186/s13195-023-01219-4)
Supplement: Supplementary file 1 — Additional file 1: Supplementary Material A. Table S1. Overview of source population (ORIMA) and comparable census demographics. Table S2. Percentage of respondents willing to undertake each dementia testing modality by age group and gender. Table S3. Percentage of respondents endorsing items pertaining to dementia testing by age group and gender. Supplementary Material B. Information pertaining to individual survey items. [file 13195_2023_1219_MOESM1_ESM.docx]

**Supplementary Material A**

**Table S1.** Overview of source population (ORIMA) and comparable census demographics.

|  | % Registered Users | % Census Data |
| --- | --- | --- |
| Population | ~452,000 | ~25million |
| Gender^a^ |  |  |
| *Male*  *Female* | 53  47 | 49  51 |
| Age Range |  |  |
| *18-24*  *25-34*  *35-44*  *45-54*  *55+* | 11  22  25  22  20 | 13  20  20  18  28 |
| State or Territory |  |  |
| *New South Wales*  *Australian Capital Territory*  *Victoria*  *Western Australia*  *Queensland*  *South Australia*  *Tasmania*  *Northern Territory* | 32  3  23  10  20  8  2  2 | 33  2  25  10  20  8  1  1 |
| Educational Attainment |  |  |
| Primary Language | English | English |

*Note.* Information from the ORIMA Pureprofile panel. Pureprofile sources new account holders (panel members) through a variety of online and offline sources including, but not limited to, internal referral programs, paid acquisition, social media, PR, search engine marketing, offline marketing and location-based registration. A number of preventative measures (e.g. account validation) are employed to detect and deter fraudulent activity.

^a^Based on census data prior to the inclusion of non-binary gender.

**Table S2.** Percentage of respondents willing to undertake each dementia testing modality by age group and gender.

|  |  | % Yes | | |  | % No | | |
| --- | --- | --- | --- | --- | --- | --- | --- | --- |
|  |  | 18-39 | 40-59 | 60+ |  | 18-39 | 40-59 | 60+ |
| Blood | Male | 44.3 | 58.5 | 72.1 |  | 55.7 | 41.5 | 27.9 |
|  | Female | 63.6 | 64.2 | 58.3 |  | 36.4 | 35.8 | 41.7 |
| Saliva | Male | 12.2 | 6.1 | 23.3 |  | 87.8 | 93.9 | 76.7 |
|  | Female | 8.1 | 15.6 | 6.3 |  | 91.9 | 84.4 | 93.8 |
| Genetic | Male | 46.6 | 46.3 | 65.1 |  | 53.4 | 53.7 | 34.9 |
|  | Female | 59.6 | 67.0 | 62.5 |  | 40.4 | 33.0 | 37.5 |
| Lumbar Puncture | Male | 12.2 | 6.1 | 23.3 |  | 87.8 | 93.9 | 76.7 |
|  | Female | 8.1 | 15.6 | 6.3 |  | 91.9 | 84.4 | 93.8 |
| Aroma | Male | 30.5 | 43.9 | 50.0 |  | 69.5 | 56.1 | 50.0 |
|  | Female | 50.5 | 48.6 | 42.7 |  | 49.5 | 51.4 | 57.3 |
| Retinal Imaging | Male | 37.4 | 42.7 | 61.6 |  | 62.6 | 57.3 | 38.4 |
|  | Female | 52.5 | 59.6 | 49.0 |  | 47.5 | 40.4 | 51.0 |
| Cognitive | Male | 43.5 | 48.8 | 72.1 |  | 56.5 | 51.2 | 27.9 |
|  | Female | 58.6 | 61.5 | 65.6 |  | 41.4 | 38.5 | 34.4 |
| Physiological | Male | 39.7 | 31.7 | 44.2 |  | 60.3 | 68.3 | 55.8 |
|  | Female | 45.5 | 46.8 | 28.1 |  | 54.5 | 53.2 | 71.9 |
| Modifiable Risk-Factors | Male | 34.4 | 26.8 | 54.7 |  | 65.6 | 73.2 | 45.3 |
|  | Female | 52.5 | 52.3 | 45.8 |  | 47.5 | 47.7 | 54.2 |

**Table S3.** Percentage of respondents endorsing items pertaining to dementia testing by age group and gender.

|  | |  | | 18-39 | | | | | |  | | 40-59 | | | | |  | | | 60+ | | | | | |
| --- | --- | --- | --- | --- | --- | --- | --- | --- | --- | --- | --- | --- | --- | --- | --- | --- | --- | --- | --- | --- | --- | --- | --- | --- | --- |
|  | |  | | Yes | | No | Unsure | | |  | | Yes | No | Unsure | | | |  | | | Yes | No | | Unsure | |
| Would choose to know dementia risk | Male | | 56.5 | | 19.8 | | | 23.7 |  | | 52.4 | | 26.8 | | 20.7 |  | | | 67.4 | | | | 19.8 | | 12.8 |
|  | Female | | 67.7 | | 17.2 | | | 15.2 |  | | 65.1 | | 13.8 | | 21.1 |  | | | 67.7 | | | | 20.8 | | 11.5 |
| Understands dementia testing possibly inconclusive | Male | | 38.2 | | 38.2 | | | 23.7 |  | | 40.2 | | 41.5 | | 18.3 |  | | | 48.8 | | | | 26.7 | | 24.4 |
|  | Female | | 42.4 | | 40.4 | | | 17.2 |  | | 36.7 | | 34.9 | | 28.4 |  | | | 46.9 | | | | 29.2 | | 24.0 |
| Would seek genetic counselling | Male | | 51.1 | | 20.6 | | | 28.2 |  | | 42.7 | | 25.6 | | 31.7 |  | | | 57.0 | | | | 14.0 | | 29.1 |
|  | Female | | 48.5 | | 25.3 | | | 26.3 |  | | 45.9 | | 16.5 | | 37.6 |  | | | 44.8 | | | | 20.8 | | 34.4 |
| Knowing risk would influence lifestyle choices | Male | | 58.0 | | 19.1 | | | 22.9 |  | | 58.5 | | 18.3 | | 23.2 |  | | | 68.6 | | | | 19.8 | | 11.6 |
|  | Female | | 76.8 | | 12.1 | | | 11.1 |  | | 65.1 | | 10.1 | | 24.8 |  | | | 68.8 | | | | 14.6 | | 16.7 |
| Knowing risk would influence future plans | Male | | 54.2 | | 22.1 | | | 23.7 |  | | 50.0 | | 24.4 | | 25.6 |  | | | 68.6 | | | | 18.6 | | 12.8 |
|  | Female | | 63.6 | | 21.2 | | | 15.2 |  | | 62.4 | | 13.8 | | 23.9 |  | | | 67.7 | | | | 14.6 | | 17.7 |
| Would pay for testing | Male | | 62.6 | | 21.4 | | | 16.0 |  | | 51.2 | | 32.9 | | 15.9 |  | | | 57.0 | | | | 30.2 | | 12.8 |
|  | Female | | 60.6 | | 26.3 | | | 13.1 |  | | 56.9 | | 25.7 | | 17.4 |  | | | 49.0 | | | | 34.4 | | 16.7 |

**Supplementary Material B**

**Section 1a. Lifestyle Habits and Disease Prevention**

*This section will ask questions about your lifestyle habits and how you feel about reducing your risk of developing health problems. There are no right or wrong answers. Your responses will help to inform scientists and medical professionals about how to better meet the needs of the community.*

1. **Do you feel adequately informed about the ways in which you can reduce your risk of developing health problems? [tick all that apply]**
   - Yes, heart disease
   - Yes, stroke
   - Yes, cancer
   - Yes, mental illness e.g. depression, anxiety
   - No, I don’t feel informed about risk-reduction for any of the above
2. **Do you feel that information about reducing your risk of developing health problems is easily available? [tick all that apply]**
   - Yes, heart disease
   - Yes, stroke
   - Yes, cancer
   - Yes, mental illness e.g. depression, anxiety
   - No, I don’t feel information about risk-reduction for any of these is easily available
3. **From which sources do you/would you seek information about reducing your risk of developing health problems? [tick all that apply]**
   - Doctor
   - Allied health professional, e.g. dietitian, physio
   - The internet/websites
   - Books or magazines
   - Organisation e.g. Heart Foundation, Cancer Council
   - Friends/Family
   - Other, **please specify ______________**
4. **Do you feel confident in your ability to apply healthy lifestyle strategies to your overall health management to help prevent serious disease? [tick all that apply]**
   - Yes, heart disease
   - Yes, stroke
   - Yes, cancer
   - Yes, mental illness e.g. depression, anxiety
   - No, I don’t feel confident in applying risk-reduction strategies for any of these
5. **Which of the following strategies are you currently applying to your overall health management? [tick all that apply]**
   - Increasing physical activity
   - Losing weight/maintaining a healthy weight
   - Healthy diet
   - Being socially active
   - Maintaining mental health
   - Keeping my brain active
6. **Do you feel that your overall likelihood of developing health problems is somewhat under your control?**
   - Yes
   - No
   - Don’t know
7. **Do you feel that risk-reduction is an important component of a healthy lifestyle?**

- Yes
- No
- Don’t know

**Section 1b. Dementia Risk-reduction**

*This section will ask questions more specifically about reducing your risk of developing* ***dementia****. There are no right or wrong answers. Your responses will help to inform scientists and medical professionals about how to better meet the needs of the community.*

1. **Do you feel adequately informed about the ways in which you can reduce your risk of developing dementia?**
   - Yes
   - No
   - Don’t know
2. **Do you feel that information about reducing your dementia risk is easily available?**
   - Yes
   - No
   - Don’t know
3. **From which sources do you/would you seek information about reducing your dementia risk? [tick all that apply]**
   - Doctor
   - Allied health professional e.g. dietician, physio
   - The internet/websites
   - Books or magazines
   - Dementia organisation e.g. Dementia Australia
   - Friends/family
   - Other, **please specify _______**
4. **Do you feel confident in your ability to apply dementia risk-reduction strategies to your overall health management?**
   - Yes
   - No
   - Don’t know
5. **Do you feel that your overall likelihood of developing dementia is somewhat under your control?**
   - Yes
   - No
   - Don’t know
6. **Do you feel that dementia risk-reduction is an important component of a healthy lifestyle?**
   - Yes
   - No
   - Don’t know
7. **Is dementia prevention currently a priority in relation to your overall health care plan?**
   - Yes
   - No
   - Don’t know

*If no, go to 14a*

**14a If no, do you intend to make dementia prevention a priority in the future?**

- - Yes
  - No
  - Don’t know

1. **Are you aware that many dementia risk-reduction strategies overlap with risk-reduction strategies for other diseases?**
   - Yes
   - No
   - Don’t know
2. **Does knowing that healthy lifestyle changes may also help you to reduce your risk of dementia make you more likely to adopt them to reduce your dementia risk?**
   - Yes
   - No
   - Don’t know

*If ‘Yes”, go to 16a*

**16a. If yes, which of the following risk-reduction strategies are you more likely to engage in to reduce your dementia risk?**

- - Increasing physical activity
  - Losing weight/maintaining a healthy weight
  - Healthy diet
  - Being socially active
  - Maintaining mental health
  - Keeping my brain active

1. **Are you fearful of developing dementia?**

- Yes
- No
- Don’t know

*If ‘Yes”, go to 17a*

**17a. If yes, is fear a strong motivator for you to implement dementia prevention strategies?**

- Yes
- No
- Don’t know

1. **Do you believe that dementia risk-reduction can be an enjoyable component of an overall healthy lifestyle?**

- Yes
- No
- Don’t know

1. **Are any of the following barriers preventing you from engaging in healthy lifestyle habits which may help to reduce your dementia risk? [tick all that apply]**

- I can’t afford to, e.g. costs of gym membership, healthy food, psychologist
- I don’t have the support I need, e.g. family commitments, carer responsibilities
- My mobility is limited, e.g., unable to exercise independently
- I don’t have time, e.g. work or study commitments
- I don’t feel safe in my home or community
- I have other health problems which are more important
- I don’t know what to do
- I lack motivation
- Other, **please specify ____________**

1. **Which of the following would make you more likely to engage in healthy lifestyle habits which may help to reduce your dementia risk? [tick all which apply]**
   - More easily accessible information
   - Greater incentive, e.g. health fund rebates, workplace programs
   - Increased access to allied health services e.g. psychologist, physio
   - More green space in which to exercise
   - Safer communities
   - Lower cost of healthy food
   - Healthy eating advice
   - Carer supports e.g. daycare, respite care
   - Community classes supporting dementia prevention e.g. dance, fitness, drama
   - Other, **please specify ___________**
   - Don’t know
2. **Do you think that the government should be providing more programs to support dementia prevention within the community?**
   - Yes
   - No
   - Don’t know
3. **If more dementia prevention programs were available within your local community, would you be likely to participate in these programs?**
   - Yes
   - No
   - Don’t know
4. **Are there any other comments you would like to make regarding your beliefs or experiences about dementia prevention?**

**Section 2a. Health Testing**

*This section will ask how you feel about testing for various health problems. There are no right or wrong answers. This information will help medical professionals and scientists to best meet the needs of the community.*

1. **At what age did you/would you feel comfortable beginning to discuss your risk of developing health problems such as cancer and heart disease with your medical professional?**
   - Young adult
   - Middle age
   - Older adulthood
2. **If you had the option, would you like to know your likelihood of developing serious health problems?**
   - Yes, cancer
   - Yes, heart disease
   - Yes, mental health issues
   - No, I do not want to know
   - Don’t know

*If ‘no’ go to 25a*

**25a If no, why not? [tick all that apply]**

- - Anxiety regarding outcome
  - Nothing you can do about it
  - Would just rather not know
  - I don’t believe it would be useful to me
  - It is not in line with my cultural or religious values
  - I don’t trust the results
  - Other, **please specify ___________**

1. **Do you feel that knowing your likelihood of developing health problems would influence your lifestyle choices?**
   - Yes
   - No
   - Don’t know

*If ‘yes’ go to 26a*

**26a If yes, in what way?**

- *More* likely to make positive lifestyle changes
- *Less* likely to make positive lifestyle changes
- Don’t know

1. **Do you feel that knowing your likelihood of developing serious health problems may influence your plans for the future?**
   - Yes
   - No
   - Don’t know
2. **Do you have concerns regarding finding out your risk of developing serious health problems in relation to any of the following areas?**
   - Privacy
   - Obtaining medical/life Insurance
   - Employment
   - Family planning
   - Relationships
   - Other, **please specify ______**
3. **If it was affordable, would you be willing to pay for testing in order to determine your risk of developing serious health problems?**
   - Yes, cancer
   - Yes, heart disease
   - Yes, mental health issues
   - No
   - Don’t know

**Section 2b. Dementia Testing**

*This section will ask about the emerging area of determining your likelihood of developing dementia. It is important to note that many of these tests are not yet available from your doctor. However, your responses will help to inform scientists and medical professionals about how to best meet the needs of the community as research continues to advance in this area.*

1. **If you had the option, would you like to know your likelihood of getting dementia?**
   - Yes
   - No
   - Don’t know

*If ‘no’ go to 30a*

**30a If no, why not? [tick all that apply]**

- Anxiety regarding outcome
- Nothing you can do about it
- Would just rather not know
- I don’t believe it would be useful to me
- It is not in line with my cultural or religious values
- I don’t trust the results
- Other

**If ‘other’ please specify _______________**

*If ‘yes’ go to 30b*

**30b If yes, would you still want to know given there are currently few treatment options available?**

- Yes
- No
- Don’t know

1. **The ability to determine your likelihood of developing dementia is still an emerging science. Are you aware that many dementia tests, now and in the future, may not give conclusive answers?**

- Yes
- No
- Don’t know

1. **Would you consider seeing a health professional who is specially trained in medical testing in order to help you decide whether to pursue dementia testing?**
   - Yes
   - No
   - Don’t know
2. **Do you feel that knowing your likelihood of developing dementia would influence your lifestyle choices?**
   - Yes
   - No
   - Don’t know

*If ‘yes’ go to 33a*

**33a If yes, in what way?**

- - More likely to make positive lifestyle changes
  - Less likely to make positive lifestyle changes
  - Don’t know

1. **Do you feel that knowing your likelihood of developing dementia may influence your plans for the future?**
   - Yes
   - No
   - Don’t know
2. **Do you have concerns regarding finding out your dementia risk in relation to any of the following areas?**
   - Privacy
   - Obtaining medical/life Insurance
   - Employment
   - Family planning
   - Relationships
   - Other

**If ‘other’ please specify ______________**

1. **Would you be willing to pay for testing in order to determine your risk of developing dementia?**
   - Yes, no matter what the cost
   - Yes, if it was affordable
   - No
   - Don’t know
2. **Which of the following assessments would you be willing to undertake in order to determine your likelihood of developing dementia? [tick all that apply]**
   - Blood test for biomarkers e.g. cholesterol, inflammation (needle into arm)
   - Saliva test (mouth swab)
   - Genetic testing (blood/saliva test)
   - Lumbar puncture (needle into spine to collect cerebral spinal fluid)
   - Aroma test (smelling a range of tubes containing various substances)
   - Retinal imaging (photograph of eyes)
   - Cognitive testing e.g. memory, problem solving ability
   - Physiological e.g. brain stimulation
   - Assessment of modifiable risk factors e.g., lifestyle, other medical conditions
3. **At what age did you/would you feel comfortable beginning to discuss your risk of developing dementia with your medical professional?**
   - Young adult
   - Middle age
   - Older adulthood

**Section 3. Dementia Knowledge**

*This section includes a few questions about what you currently know about dementia. These questions are designed to show us how well information about dementia is being communicated with the community.*

1. **Which of the following best describes your connection to dementia?**
   - I am a person diagnosed with mild cognitive impairment
   - I am a person diagnosed with dementia
   - I am a carer of a person with dementia
   - I have a relative/friend with dementia
   - I am a health professional
   - I have no direct connection to dementia
   - Other, **please specify __________**
2. **Of which of the following forms of dementia are you aware?**
   - Alzheimer’s disease
   - Vascular dementia
   - Frontotemporal dementia
   - Lewy body dementia
   - Other, **please specify ____________**
3. **Which of the following symptoms do you associate with a dementia diagnosis? [tick all that apply]**
   - Memory issues and forgetfulness
   - Difficulty planning and organising
   - Getting lost
   - Loss of social skills
   - Losing interest in things once enjoyed
   - Showing less emotion towards loved ones
   - Perceptual difficulties e.g. vision changes, loss of sense of smell
   - Other, **please specify_______**
4. **To the best of your knowledge, from what age do lifestyle and other risk factors begin to contribute to one’s likelihood of developing dementia?**
   - Early life
   - Young adult
   - Middle age
   - Older adulthood
5. **Do you consider dementia a natural part of ageing?**

- Yes
- No
- Don’t know

**Section 4. Demographics**

*This section will ask a little about you to help us compare how well information is reaching different parts of the community.*

1. **Age range**

- 18-39
- 40-59
- 60 or over

1. **What is your gender?**

- Male
- Female
- Other, **please specify_______________**
- Prefer not to answer

1. **What is your postcode?**
2. **How many years of full-time education have you completed?**

*___years*

1. **What is the highest qualification that you have completed?**

- *School certificate/Year 10*
- *Higher school certificate (HSC)/Year 12*
- *Trade certificate/apprenticeship*
- *Technical certificate I – IV*
- *Diploma*
- *Advanced diploma/Associate degree*
- *Bachelor degree*
- *Bachelor Honours/Graduate Certificate/Graduate Diploma*
- *Masters degree*
- *Doctorate*

1. **Are you currently employed?**

- Yes
- No

*If yes go to 49a.*

**49a If you answered Yes, what is your occupation?**

*If no go to 49b*

**49b If you answered No, are you currently seeking employment?**

- - Yes
  - No

1. **Do you live alone?**
   - Yes
   - No
   - Prefer not to answer

*If no go to 50a.*

**50a. If you answered No, who else lives with you at home?**

- Spouse/partner
- Other relative
- Other**, please specify____**

1. **Were you born in Australia?**

- Yes
- No

*If No go to 50a and 50b*

**46a. In what country were you born?**

**46b. How many years have you lived in Australia?**

1. **Do you identify as Aboriginal or Torres Strait Islander?**

- Yes
- No

1. **Do you identify with any other cultural heritage?**
   - Yes
   - No

*If Yes go to 53a*

**53a. If Yes, please specify**_______________

1. **Do you speak a language other than English at home?** **(If more than one language, please indicate the one that is spoken most often).**
   - No, English only
   - Yes, Arabic

- Yes, Mandarin
- Yes, Cantonese
- Yes, Vietnamese
- Yes, Greek
- Yes, Italian
- Yes, Tagalog (Filipino)
- Yes, Hindi
- Yes, Other, **please specify____________________**

1. Are there any other comments you would like to make regarding the topics covered throughout this survey?

**Thank you for participating in this survey. Your time and corporation are greatly appreciated.**
